# Function of *Thelenota ananas* saponin desulfated holothurin A in modulating cholesterol metabolism

**DOI:** 10.1038/s41598-018-27932-x

**Published:** 2018-06-22

**Authors:** Qi-an Han, Kaifeng Li, Xiuping Dong, Yongkang Luo, Beiwei Zhu

**Affiliations:** 1 Beijing Advanced Innovation Center for Food Nutrition and Human Health, College of Food Science and Nutritional Engineering, China Agricultural University, Beijing, 100083 China; 2 School of Food Science and Technology, Dalian Polytechnic University, National Engineering Research Center of Seafood, Dalian, 116034 China

## Abstract

This work was designed to separate and purify the saponin from *Thelenota ananas* with the highest anti-cholesterol ability using multiple chromatography and mass spectrometry analyses, and to systematically investigate the effect of the *Thelenota ananas* saponin on cholesterol metabolism in oxidized low-density lipoprotein (ox-LDL) induced macrophage foam cells. Desulfated holothurin A (desHA), which was finally identified as the targeted saponin with the highest activity in decreasing low-density lipoprotein-cholesterol (LDL-C), markedly inhibited the formation of foam cells derived from macrophages based on Oil Red O staining. In addition, desHA significantly blocked the synthesis of fatty acid synthetase while promoted intracellular cholesterol efflux. Furthermore, desHA inhibited the effects of ox-LDL on macrophage mRNA expression, which enhanced the level of 3-hydroxy-3-methylglutaryl coenzyme A reductase (HMG-CoAR) and suppressed the expression of SR-BI, ABCA1 and ABCG1, which further increased the levels of extracellular cholesterol and triglyceride. Blocking AKT and AMPK pathway and LXR synthesis revealed that desHA also regulated the contents of HMG-CoAR and eNOS via LXR/AKT/AMPK pathway. Thus, desHA played an essential role in cholesterol efflux and synthesis, which indicated desHA and *Thelenota ananas* are valuable resources to exploit new functional food and nutraceuticals.

## Introduction

Saponins are the primary glycosylated secondary metabolites found in many major crops and certain sea foods, such as the sea cucumber, starfish and alcyonacea^1^. *Thelenota ananas* is an edible cucumber species popular in South China and some Southeast Asian countries, which contains the most abundant saponins both in the body wall and alimentary canal. The common structure of saponins has been identified as combination of triterpenoid or spirosterane sapogenins and oligosaccharides. Notably, active compositions of sea cucumber saponins have been clarified by using silica-gel column chromatography, high performance liquid chromatography (HPLC) and high performance centrifugal partition chromatography (HPCPC) and other newly developed technologies, including holothurin A1 (HA), holothurin B (HB), 24-dehydroechinoside A (DA), echinoside A (EA), argusides, coustesids, holothurinosides, impatiensides, marmoratoside A and philinopsides^2–10^.

Sea cucumber was the most special marine animal found existing some saponins with a lot of remarkable bioactivities in inhibiting tumor and inflammation, promoting hemolysis, and modulating circadian clock^11–16^. Moreover, sea cucumber saponins helped regulate cholesterol metabolism and further alleviated the development of obesity, hyperlipidaemia and diabetes, revealing that sea cucumber saponins potentially resistant against atherosclerosis, which is a chronic inflammatory disease induced mainly by lipid accumulation on middle and large vessels^17–19^. It was reported that saponins prevented atherogenesis mainly by regulating liver X receptor alpha (LXRα), ATP binding cassette transporter A1 (ABCA1) expressions and suppressing 3-hydroxy-3-methylglutaryl coenzyme A reductase (HMG-CoAR) activity and further lowering cholesterol and triglyceride concentration^20–22^. However, systematic and comprehensive studies are still needed to elucidate the mechanism.

Therefore, the objective of the study was to screen and identify the *Thelenota ananas* saponin with the highest activity of lowering cholesterol. The target saponin was prepared using differential column chromatography, and the structure was subsequently determined by HPLC-mass spectroscopy (MS). The changes in key signal pathways involved in cholesterol metabolism were then detected to illuminate the bioactivity of sea cucumber saponin in modulating cholesterol metabolism *in vitro*, which may provide further theoretical support regarding the anti-atherosclerotic role of *Thelenota ananas* saponin.

## Results and Analysis

### Purification and identification of *Thelenota ananas* saponin

**Figure 1:**
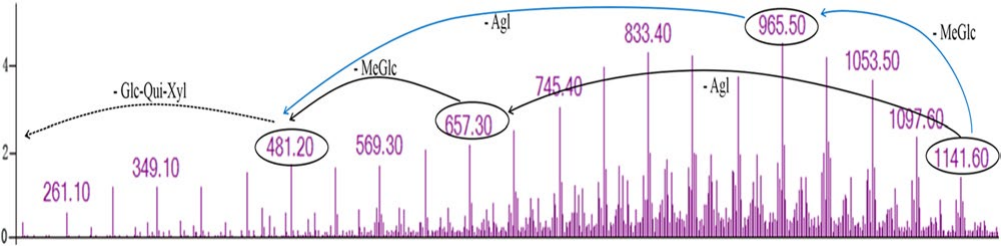
The collision-induced fragmentation patterns of compound 3 separated by semi-preparative HPLC. Blue and Black arrows are two possible fragmentation patterns. Note: Agl is short for Aglycone, MeGlc is short for 3-O-methylglucose, Glc is short for Glucose, Qui is short for Quinovose, Xyl is short for Xylose.

Crude *Thelenota ananas* saponins (29.8 g) were obtained using alcohol-extraction and liquid-liquid extraction, and were qualitatively determined using a foam test, precipitation reaction and Molisch reaction (data not shown). First, two fractions of the saponins were collected via the AB-8 macroporous resin column, i.e., 30% ethanol-eluting portion and 70% ethanol-eluting portion. Both portions decreased the content of LDL-C with the rates of 11.5% and 27.7%, respectively (data not shown). Thus, the 70% ethanol-eluting portion (16.1 g) was continuously purified using a Sephadex LH-20 column to obtain 8 fractions, and Fraction 8 (137.6 mg) exhibited excellent cholesterol decreasing activity (66.7%, data not shown). As fraction 8 was subjected to a semi-preparative HPLC, compound 1 (retention time 11.869 min), compound 2 (retention time 13.998 min), and compound 3 (retention time 16.320 min) were finally acquired, among which compound 3 (25.1 mg) possessed the highest content and anti-cholesterol properties (Supplementary Figure S1). Then, the results of the HPLC-MS analyses revealed that the molecular mass of compound 3 is 1119 Da, which is the same with desulfated holothurin A (NCBI PubChem CID: 3038027, CAS NO. 11060-73-4, desHA). DesHA has been previously detected in sea cucumber *Holothuria forskali*, also known as nobiliside 2A^23^. Therefore, it was inferred that the structure of compound 3 was 3-O-[3-O-methyl-β-D-glucopyranosyl-(1 → 3)-β-D-glucopyranosyl-(1 → 4)-β-D-quinovose-(1 → 2)-β-D-xylopyranosyl]-22,25-epoxyholost-9-ene-3β,12α,17α-trihydroxy. According to this, there might be two sequences of fragmentation of compound 3 at *m/*z 1141.6, showing the consecutive losses of 3-O-methylglucose, aglycone, and glucose-quinovose-xylose sodium or consecutive losses of the aglycone, 3-O-methylglucose, and glucose-quinovose-xylose sodium from the mass-selected compound 3 (Fig. 1). These findings enabled the identification of the *m/*z 1141.6 ions as desHA.

### Effect of desHA on the viability of RAW264.7 cells

**Figure 2:**
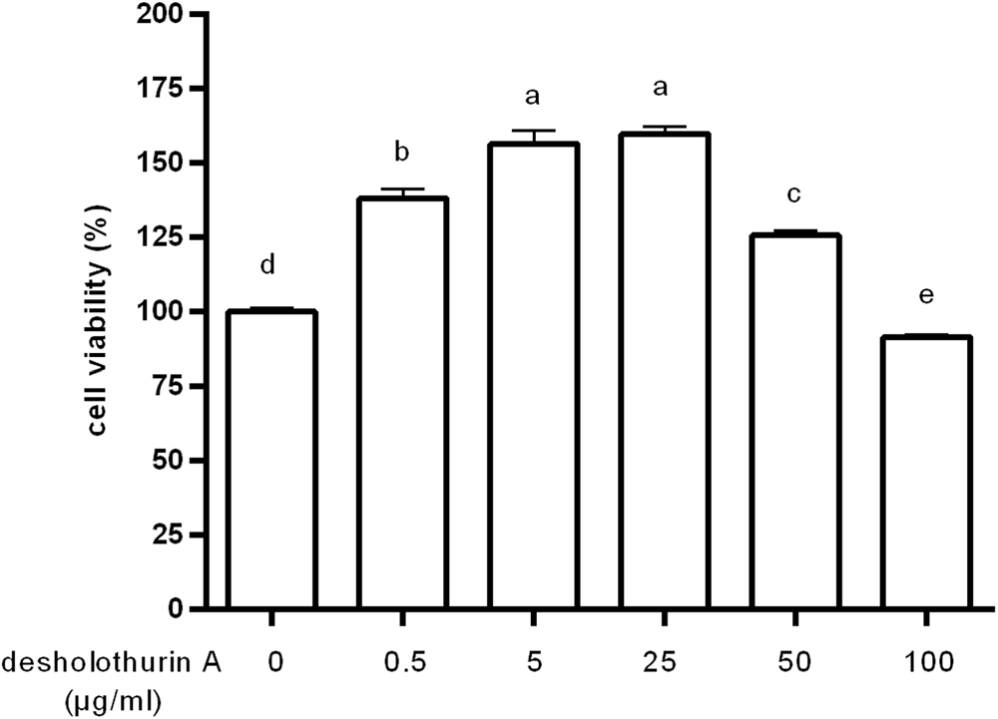
The effect of desHA on RAW264.7 cell viability. Results were presented as mean ± SD of three independent experiments. Values having different superscripts are significantly different, p < 0.05 (same to the below figures).

DesHA imapcted the cell viability of macrophage depended on its concentrations (Fig. 2). Specifically, 0.5 to 25 μg/mL of desHA prepared from *Thelenota ananas* gradually increased cell proliferation, while higher concentrations of saponin induced a weaker increase in the viability of RAW264.7 cells. Thus, low concentrations of 1, 3 and 5 μg/mL were used in the following experiments.

### DesHA inhibited the formation of foam cells derived from macrophage

**Figure 3:**
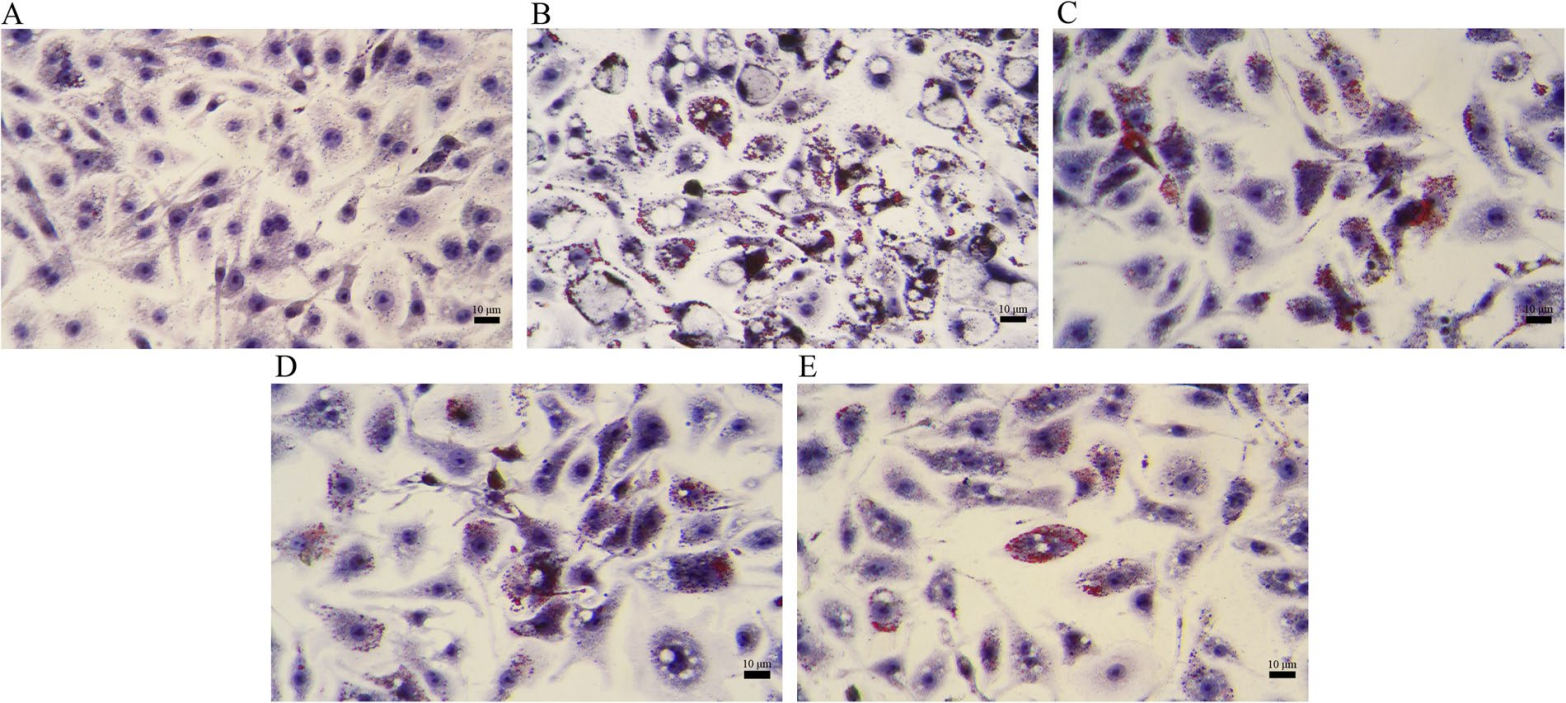
The effect of desHA on macrophage-derived foam cells formation with a magnification of ×400. (**A**) control group, (**B**) ox-LDL group, (**C**) ox-LDL + 1 µg/mL desHA group, (**D**) ox-LDL + 3 µg/mL desHA group, (**E**) ox-LDL + 5 µg/mL desHA group.

Figures 3A,B illustrate that ox-LDL apparently induced a larger proportion and size of foam cells than those in the control group, which primarily maintained the normal macrophages. However, desHA gradually decreased the lipid intake of macrophages and the formation of foam cells with increasing concentration (Fig. 3C–E).

### Effect of desHA on fatty acid synthetase (FAS), triglyceride (TG) and total cholesterol (TC)

**Figure 4:**
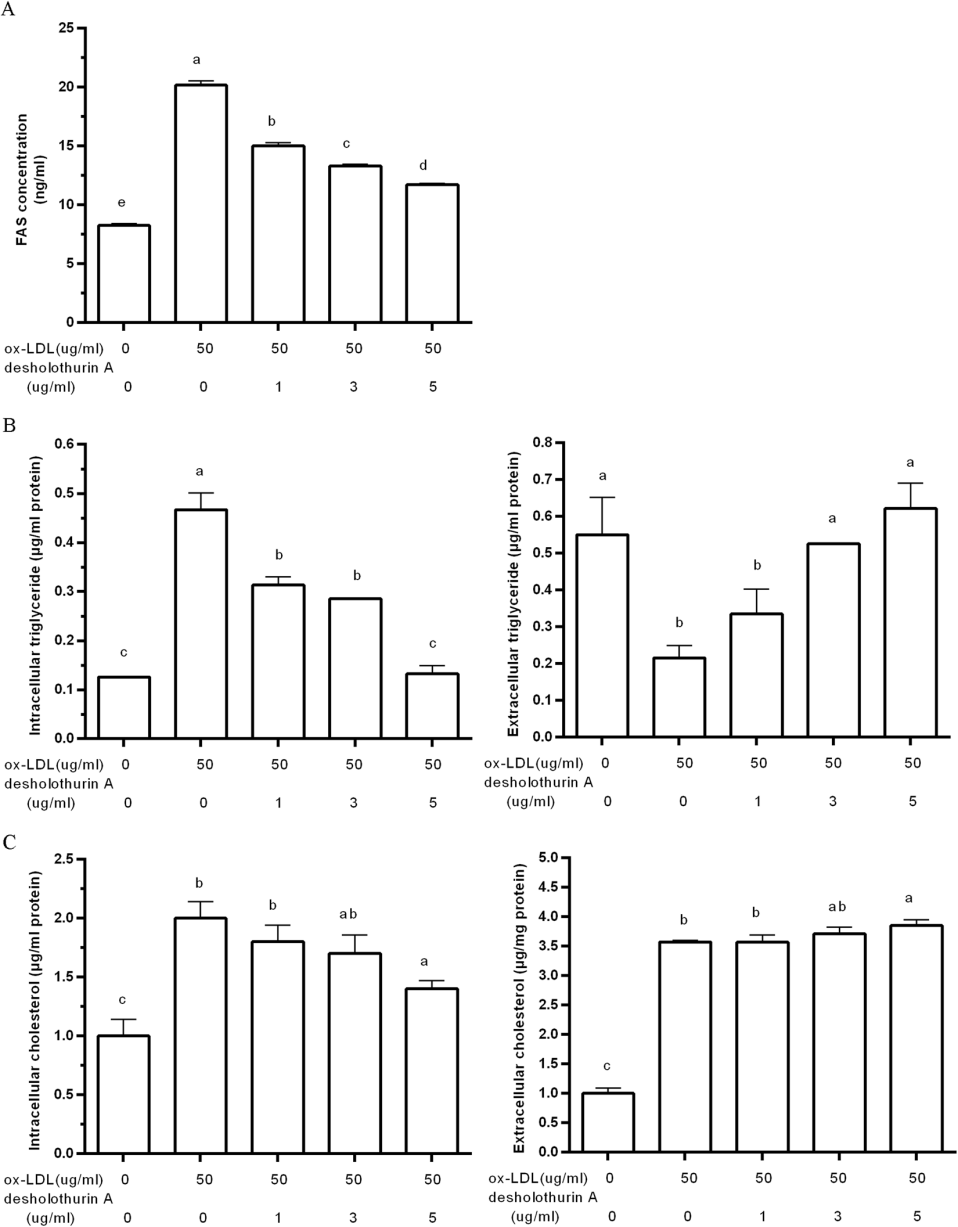
The effect of desHA on the productions of FAS (**A**), TG (**B**) and TC (**C**) in RAW264.7 cells.

Ox-LDL has been shown to induce the transformation of RAW264.7 cells into foam cells. Thus, we detected the influence of desHA on ox-LDL induced foam cells. DesHA inhibited the content of ox-LDL-induced FAS with a dose-dependent manner (Fig. 4A). Figure 4B,C demonstrate that desHA repressed the intracellular triglyceride and cholesterol levels. Meanwhile, desHA increased the contents of extracellular triglyceride and cholesterol, thereby promoting the efflux of both triglyceride and cholesterol.

### Effect of desHA on the expression of HMG-CoAR, SR-BI, ABCA1 and ABCG1

**Figure 5:**
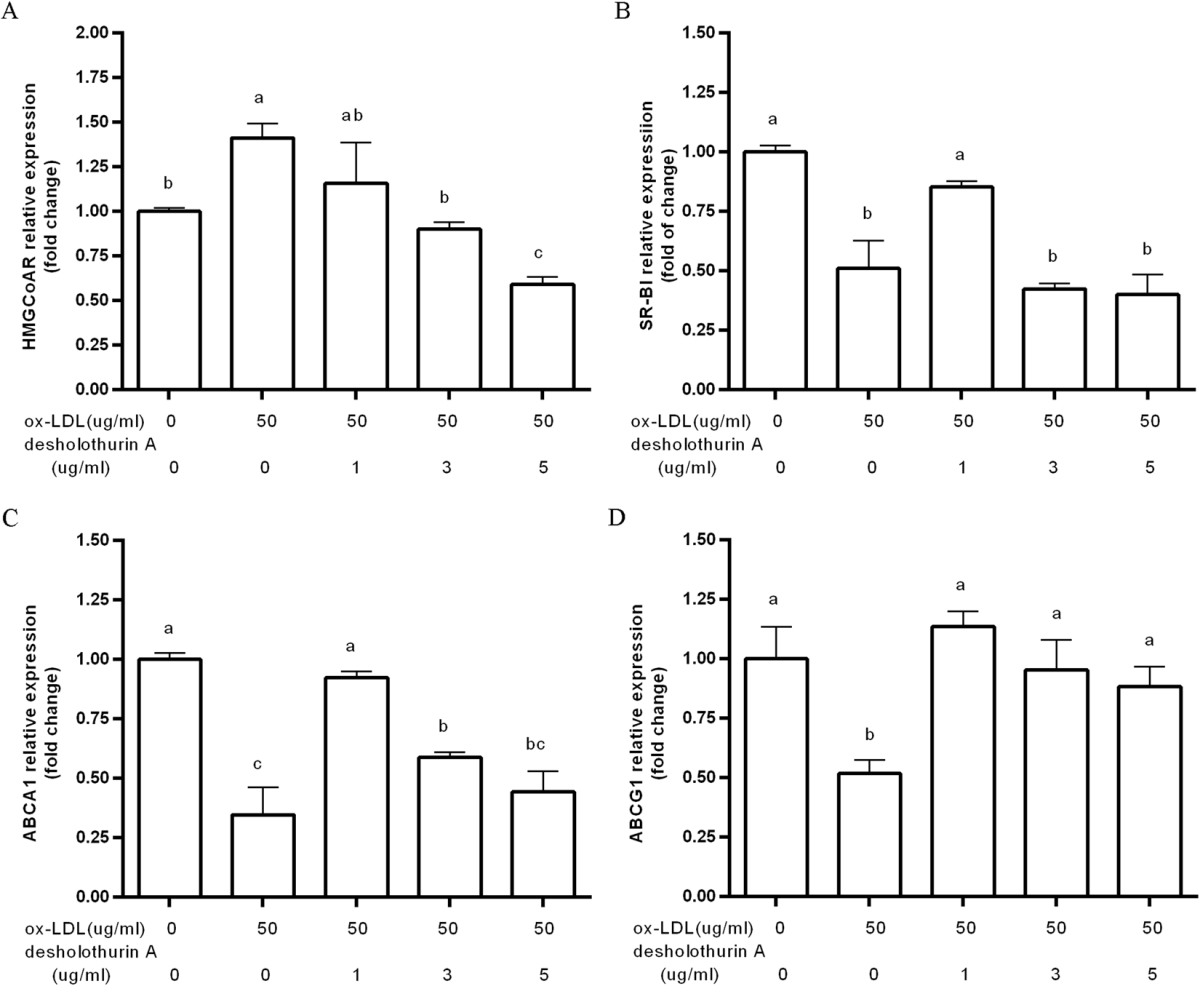
The effect of desHA on the expressions of cholesterol metabolism related factors HMG-CoAR (**A**), SR-BI (**B**), ABCA1 (**C**) and ABCG1 (**D**).

Figure 5 shows that the mRNA expression of HMG-CoAR elevated by 1.5-fold, while the expressions of SR-BI, ABCA1 and ABCG1 were markedly down-regulated by ox-LDL compared with that in the control group. In contrast, desHA significantly reversed the influence of ox-LDL. Specifically, the HMG-CoAR level gradually declined with increased desHA concentration (Fig. 5A). Compared with the ox-LDL treatment, the mRNA expressions of SR-BI, ABCA1 and ABCG1 treated by 1 μg/mL desHA were up-regulated by 2-fold, 2.4-fold and 2.3-fold, respectively (Fig. 5B,C,D).

### DesHA modulated AKT and AMPK signal pathways via regulating LXRα in RAW264.7

**Figure 6:**
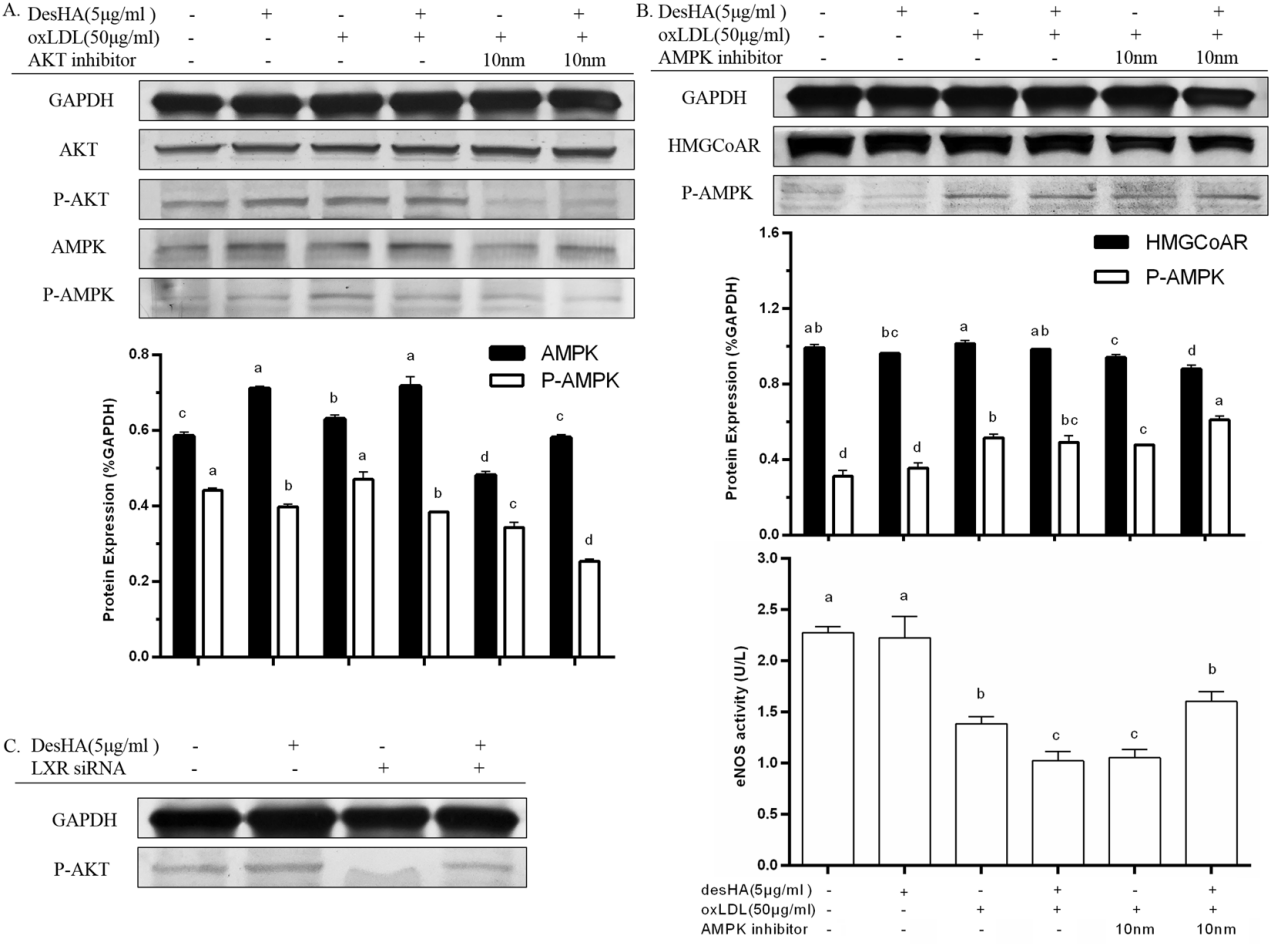
The cell signal pathways mediated by desHA in cholesterol metabolism. (**A**) The changes of AKT, P-AKT, AMPK and P-AMPK levels treated by AKT inhibitor with or without desHA. (**B**) The changes of P-AMPK and its downstream protein factors treated by AMPK inhibitor with or without desHA. (**C**) The changes of P-AKT expression treated by LXR siRNA with or without desHA.

DesHA played an essential role in regulating the phosphorylated proteins of AKT and AMPK in ox-LDL induced macrophage foam cells (Fig. 6A). Obviously, an increment of phosphor-AKT and AMPK accompanied by suppressed phosphor-AMPK were observed under the treatment of desHA with or without ox-LDL.

Figure 6A also indicates that no significant change of AKT total protein content was observed with addition of AKT inhibitor MK-2206 2HCl, however, phosphor-AKT was almost entirely inhibited by AKT inhibitor and then being restored by desHA. Besides, the expressions of AMPK and phosphor-AMPK were changed when blocking AKT signal pathway, but desHA inhibited the transform of AMPK to phosphor-AMPK with or without the treatment of AKT inhibitor, which showing an important regulation effect of desHA to keep the homeostasis of AMPK content via AKT signal pathway.

Furthermore, the treatment of AMPK inhibitor Dorsomorphin markedly decreased phosphor-AMPK content which was then reversed by desHA (Fig. 6B). It also illustrates that blocking AMPK signal pathway decreased the levels of HMG-CoAR. Meanwhile, desHA suppressed the HMG-CoAR expression with or without the addition of AMPK inhibitor. In addition, the activity of eNOS was suppressed both by Dorsomorphin and desHA in comparison with the one treated by ox-LDL, but desHA finally enhanced the activity of eNOS in cells under the ox-LDL and Dorsomorphin treatment.

Figure 6C demonstrates that LXR promoted AKT activation because little expression of phosphor-AKT was accordingly found by knockdown LXR. However, desHA significantly revised the inhibition of phosphor-AKT. Consequently, desHA might exhibit its decreasing-cholesterol property by modulating LXR/AKT/AMPK pathway.

## Discussion

Different extraction methods were used to identify saponins with different structures, and the discovery of new structures of saponins with various bioactivities in the sea cucumber have attracted more attention. In this work, desHA was separated from *Thelenota ananas* for the first time confirmed by the positive tandem ESI-MS results.

According to the latest statistics, scholars have isolated more than 700 types of sea cucumber saponins from nearly hundreds of species of sea cucumber, including *Aspidochirotida: Holothuriidae, Aspidochirotida: Stichopodidae, Dendrochirotida: Cucumariidae*^24^. HA and HB were identified in several sea cucumber *Holothuria*^25^. Friess, *et al*.^26^ initially discussed the desulfated derivate of HA, namely, holothurin A, desulfo- or desHA, purified from the sea cucumber *Actinopyga agassizi Selenka*, in terms of the mechanism of the saponin irreversible inactivation of the ganglion and the survival of ganglionic excitability. Subsequently, desHA was detected in *Bohaadschia argus, Holothuria forskali*, *Holothuria leucospilota, Holothuria nobilis* and *Pearsonothuria graeffei*^27^.

Because of the abundant resources in the ocean, much attention has been paid to exploiting new active substances with extended application in nutraceutical, cosmeceutical, medicinal and pharmaceutical products. Although, other active substances from *Thelenota ananas*, such as fucoidan, fucosylated chondroitin sulfate, and glycosaminoglycan, have gained more attention than saponins because of their abundant content and multiple functions^28–31^. Generally, desHA is secreted as a chemical defense when sea cucumbers are stressed^32^. The degrading-cholesterol effect of desHA has not been discussed thus far. The functions of saponins in *Thelenota ananas* and other marine animals were also attractive to investigate in depth.

On one hand, the structures of saponins prepared from the sea cucumber are different from those of plant saponins. Lanostane-type triterpene glycosides (e.g. holostane types, including HA and HB), which are the most important secondary metabolites of sea cucumbers, occupied a large proportion of the purified saponins^33–35^. Particularly, the presence of an acetoxy group plays a significant role in the bioactivity, including the induction of caspase, apoptosis, cytotoxicity, and the anti-cancer, anti-fungal and anti-bacterial activities^36^. However, few studies regarding the species and bioactivities of saponins from *Thelenota ananas* have been performed.

On the other hand, several identified saponins have been studied thoroughly to understand the underlying machenism of anti-atherosclerosis effects, including panax notoginseng saponin ginsenoside-Rd^37^, reinioside C from the the root of *Polygala aureocauda* Dunn^38^, buddlejasaponin IV extract of *Pleurospermum kamtschaticum*^39^, and sea cucumber saponin EA^40^. The sulfated holothurin A prepared from *Pearsonothuria graeffei* was demonstrated to significantly reduce the adipose tissue weight in C57BL/6 mice, thereby exhibited an anti-obesity effect in male Wistar rats^9^. It seems that both crude extraction of saponin and single saponin mentioned above may decrease the concentrations of plasma cholesterol, triglyceride in animal model, therefore may play an important role in preventing cholesterol dysfunction related diseases. By far, few study concerned on the mechanisms of single saponin working in cell model to further elucidating the anti-atherosclerosis activity of the sea cucumber saponin.

**Figure 7:**
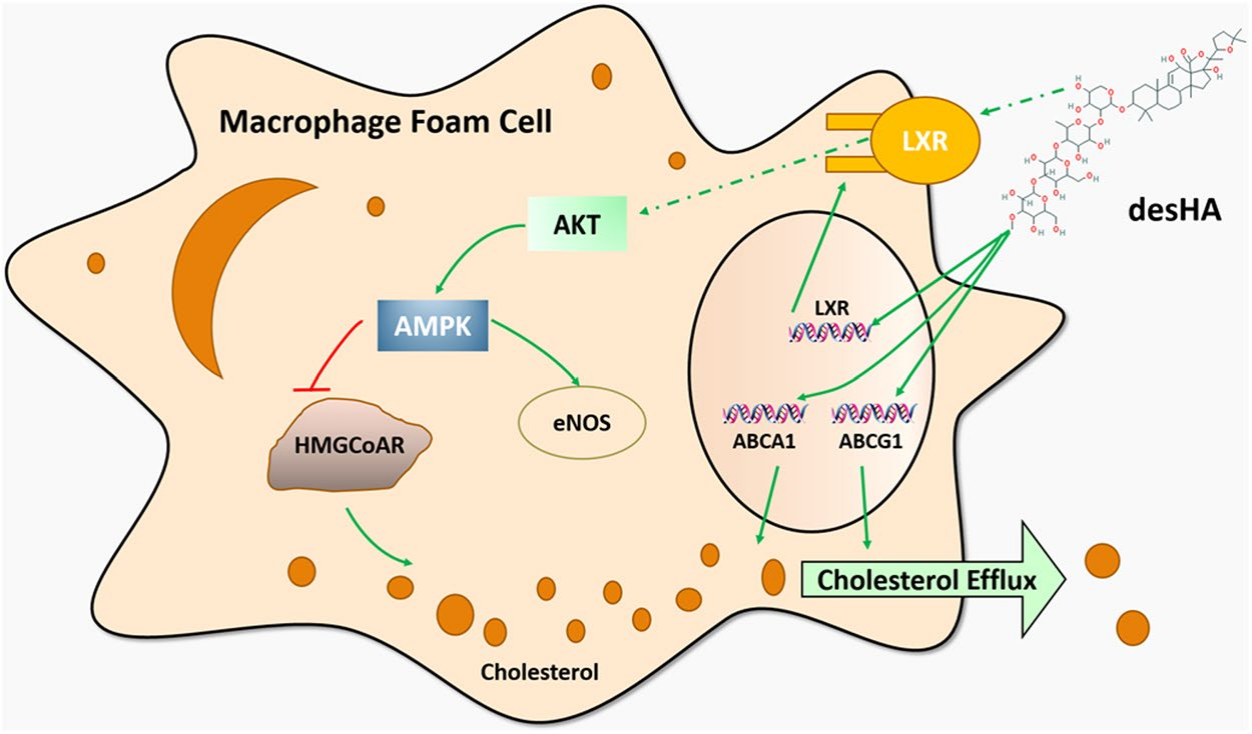
The molecular mechanism of desHA on regulating cholesterol metabolism in macrophage foam cells.

Based on these results, the underlying mechanism of the mediation effects of desHA to cholesterol content was examined in the present study, and the results indicated that desHA also showed excellent bioactivity in repressing the synthesis of cholesterol and promoting intracellular cholesterol efflux by regulating the mRNA expression of HMG-CoAR, SR-BI, ABCA1 and ABCG1. Our results also showed that desHA took part in the regulation of cholesterol metabolism via LXR/AKT/AMPK signaling pathway (Fig. 7), but there would be other signaling pathways to discover in the following study. Furthermore, most of the mechanisms of saponins effect on cholesterol metabolism and atherosclerosis progression was confirmed in cell models and direct proof in animals and human body is still need. To date, there are several mechanisms available to explain the activity of saponins apart from the regulation of cholesterol metabolism and lipase activity this work focused on, including the ability to form insoluble complexes with cholesterol and to interfere with micelle formation and bile acid metabolism^41^. And this called on researchers to further investigate the cardiovascular protection of saponins in animal models and unearth the comprehensive mechanisms.

Since saponins exhibit various activities, reflecting the surfactant properties of these compounds, it is necessary to further investigate the relationship between the activity of desHA and its structure in following study.

Overall, *Thelenota ananas* is a marine animal with a high food and potential medicinal value, and the saponins of this species possess promising multiple biological activities. This work discussed the effects of desHA on lowering cholesterol, showing that desHA is of great interest due to its potential usefulness as a food ingredient, dietary supplement or food additive for preventing certain cardiovascular diseases that are associated with abnormal cholesterol levels.

## Materials and Methods

### Materials

*Thelenota ananas* was obtained from Guangxi Simaier Co. Ltd. (Guangxi, China), and generally 5–6 organisms per 500 g were identified as superior dried sea cucumber. AB-8 macroporous resin (Qingdao Marine Chemical Co. Ltd., Qingdao, Shandong, China) and Sephadex LH-20 (GE Healthcare Bio-Sciences AB, Uppsala, Sweden) were used for column chromatography. The chemicals were of analytical grade.

The semi-preparative HPLC separation was performed on a 1260 Infinity HPLC system (Santa Clara, CA) with a C18 column (150 × 10 mm i.d., 120 Å, S-5µm). HPLC-MS analyses were performed using Agilent 6460 liquid chromatography triple quadrupole mass spectrometry (Milford, MA).

### Extraction and identification of *Thelenota ananas* saponins

The procedure was made references to Van Dyck, *et al*. and Zhu^42,43^. Dry *Thelenota ananas* (956 g) were first rehydrated by ultrapure water soaking for two days. After the homogenization, the crude saponins of *Thelenota ananas* were extracted three times using cold 70% ethanol. The combined and concentrated 70% ethanolic extraction was suspended in deionized water and successively extracted using petroleum ether and n-butanol. The n-butanol portion was concentrated by evaporation under a reduced pressure and dried by vacuum freeze-drying. Subsequently, 0.5 g/mL *Thelenota ananas* saponins was loaded and purified on a pre-treated AB-8 macroporous resin column (30 × 1000 mm) and successively eluted with 600 mL distilled water, 30% v/v, 70% v/v and 100% v/v ethanol solution at a flow rate of 3 mL/min. After rotating evaporation and freeze-drying, the collected eluent was obtained as a crude *Thelenota ananas* saponin, and the content of low-density lipoprotein-cholesterol (LDL-C) was determined by cell experiments. The 70% ethanol fraction was isolated on a Sephadex LH-20 column (10 × 500 mm) by isocratic elution with 50% to identify the best fraction with the highest cholesterol-degrading activity, which was further purified on semi-preparative HPLC with a gradient elution comprising acetonitrile and water (2 mL/min, 205 nm). The obtained compound was finally identified by the HPLC-MS analyses using an Agilent mass spectrometer. Mass data were obtained using electrospray ionization in positive ion mode.

### Detection of the cholesterol-degrading activity of saponins

The RAW264.7 macrophage cell line (China Infrastructure of Cell Line Resource, Beijing, China) was used to evaluate the effect of saponins on cholesterol metabolism. The cells were incubated in the Gibco® DMEM, containing 10% Gibco® fetal bovine serum (North America, Thermo Fisher Scientific Inc., Waltham, USA), maintained at 37 °C under a humidified 5% CO_2_ environment. The homogeneous cell suspension (1 × 10^5^/mL) were cultured in a 12 well-culture plate for 24 h. The plate was treated with different fractions of *Thelenota ananas* saponins (filtration by 0.45 μm filter membrane) for 24 h at 37 °C. The control group was treated with an equal volume of serum-free culture medium. Then, the supernatant was collected and used to detect the content of LDL-C via the LDL-C assay kit (Nanjing Jiancheng Bioengineering Institute, Nanjing, China).

### Determination of the cell viability of RAW264.7 treated with *Thelenota ananas* saponin

The RAW264.7 cell suspension (200 μL 1 × 10^5^ cell/mL) were uniformly inoculated in a 96-well cell culture plate overnight. The cells in the experimental groups were treated with different concentrations of the identified saponin from *Thelenota ananas* (0.5, 5, 25, 50, 100 μg/mL) for 6 h, and the cells in the control group were incubated in the serum-free culture medium containing 0.1% DMSO, while the blank group included only the cell culture (containing 0.1% DMSO) without cells. Each group was replicated five times. After the treatments, the cells were washed three times with ice-cold PBS, and then, 20 μL of the CCK-8 solution (Beijing Solarbio Science & Technology Co. Ltd., Beijing, China) was added to each well for 3 h. The absorbance was determined at OD570 nm using a Model 680 spectrophotometer.

### Determination of macrophage foam cell formation

In total, 1 × 10^5^/mL of RAW264.7 cells were incubated in a 6-well cell culture plate overnight. The experimental groups were stimulated with 50 μg/mL of ox-LDL. (Guangzhou Yiyuan Biotech. Co. Ltd., Guangzhou, China) for 24 h, and the control group was treated with serum-free culture medium containing 0.1% DMSO. Subsequently, the precipitate was removed, and the experimental groups were sequentially treated with or without the identified saponin at the final concentrations of 1, 3, 5 μg/mL for another 6 h. To visualize the cellular neutral lipid accumulation, the cells were fixed in 4% cold paraformaldehyde solution for 15 min, and the intracellular lipid droplet and cytomembrane were separately stained with 1% Oil Red O (in 60% isopropanol) and methylene blue at 37 °C for 10 min. The cell staining of cells was examined at 400 × magnification under an inverted microscope (Diaphot 200, Nikon, Garden City, NY).

### Quantitation of FAS, TG and TC influenced by the *Thelenota ananas* saponin

The experiment was performed as described above. According to the manufacturer’s instruction for the FAS, TG and TC detection kits (Nanjing Jiancheng Bioengineering Institute, Nanjing, China), the cell medium was collected by centrifugation, and the cells were lysed by 1% Triton X-100. Both the supernatant and the cells were used to determine the contents of the target indices.

### Genes expression involved in cholesterol metabolism influenced by the identified saponin

According to the manufacturer’s instructions for the RNA extraction kit (Tiangen Biotech Co. Ltd., Beijing, China), total RNA were extracted and the first chain of cDNA was synthesized using a reverse kit. Real-time quantitative PCR (RT-PCR) was used to detect the expression of HMG-CoAR, SR-BI, ABCA1 and ABCG1. The expression of the target genes was normalized to glyceraldehyde-3-phosphate dehydrogenase (GAPDH). The primers were following: GAPDH-F, *5′-AGGCCGGTGCTGAGTATGTC-3′*, GAPDH-R*, 5′-TGCCTGCTTCACCACCTTCT-3′*, HMGR-F, *5′-GCTTGGCCTCCATTGAGAT-3′*, HMGR-R, *5′-ATGCATCCGGAAAAGTCTTG-3′*, SRBI-F, *5′-AGGGATAGGGTTGGAGTCAGC-3′*, SRBI-R, *5′-CGTTGTAATGGAAGCCAGAGG-3′*, ABCA1-F, *5′-AAGCCAAGCATCTTCAGTTC-3′*, ABCA1-R, *5′-CCATACAGCAAGAGCAGAAGG-3′, ABCG1-F, 5′- ATACAGGGGAAAGGTCTCCAAT-3′, ABCG1-R, 5′-CCCCCGAGGTCTCTCTTATAGT-3′*.

### Determination of cholesterol-controlling signal transduction pathways by western blot assay

To explore the possible cholesterol-controlling transduction pathway of the identified saponin, cells were pre-incubated with 50 μg/mL ox-LDL for 24 h, and further separately treated with 10 nM MK-2206 2HCl (an inhibitor of AKT pathway), 5 μg/mL *Thelenota ananas* saponin and the mixture of both for another 24 h. Cells containing serum-free culture medium was set as the control group. Total protein of each treatment group was extracted by the addition of M-PER™ Mammalian Protein Extraction Reagent (Thermo Fisher Scientific Inc., Waltham, USA) and high speed centrifugation at 4 °C. Then protein concentration of the supernatant was determined and adjusted to the same level. The expressions of AKT, phosphor-AKT, AMPK, phosphor-AMPK levels were analyzed by Western Bolt assay. Rabbit polyclonal antibody to GAPDH antibody was set as the internal standard and anti-rabbit secondary antibody at 1:200 were used for immunoblotting analysis. The protein was visualized with a BCIP/NBT Alkaline Phosphatase Color Development Kit (Beyotime, Haimen, China).

In addition, with the same treatment of ox-LDL, cells were co-cultured with 10 nM AMPK inhibitor Dorsomorphin, 5 μg/mL identified saponin and the mixture of both for 24 h. Other treatments were the same as above. The protein levels of phosphor-AMPK and HMG-CoAR were determined subsequently. Besides, the supernatant was collected to detect the activity of eNOS using ELISA Kit (Sangon Biotech, Shanghai, China).

### Effect of LXR siRNA on cholesterol efflux regulation

To investigate the effect of LXR siRNA on cholesterol metabolism and related protein expression, the LXR siRNA was transfected into cells by Lipofectamine® RNAiMAX transfection reagent (Thermo Fisher Scientific Inc., Waltham, USA), then the identified saponin was added to the cells. The protein of each group was extracted and phosphor-AKT level was determined by Western blot assay.

### Statistics

Significance differences between the means were assessed using One-Way ANOVA (SPSS 19.0, SPSS Inc., Chicago, USA). P < 0.05 was considered statistically significant.

## Electronic supplementary material

Supplementary information
