# Supplementary material for: Function of Thelenota ananas saponin desulfated holothurin A in modulating cholesterol metabolism
Source: Sci Rep. 2018 Jun 22;8:9506. doi: 10.1038/s41598-018-27932-x (PMC6014995; doi:10.1038/s41598-018-27932-x)
Supplement: Supplementary file 1 — Supplementary information [file 41598_2018_27932_MOESM1_ESM.pdf]

Function of *Thelenota ananas* saponin desulfated holothurin A in modulating cholesterol metabolism

Qi-an Han [1], Kaifeng Li [1], Xiuping Dong [2], Yongkang Luo [1*], Beiwei Zhu [1, 2*]

[1] Beijing Advanced Innovation Center for Food Nutrition and Human Health, College of Food Science and Nutritional Engineering, China Agricultural University, Beijing 100083, China

[2] School of Food Science and Technology, Dalian Polytechnic University, National Engineering Research Center of Seafood, Dalian 116034, China

Corresponding author:

Beiwei Zhu, Email: zhubeiwei@163.com, Tel.: +86(411)86323262, Fax: +86(411)86323262;

Yongkang Luo, Email: luoyongkang@263.net, Tel.: +86(10)62737385, Fax: +86(10)62737385

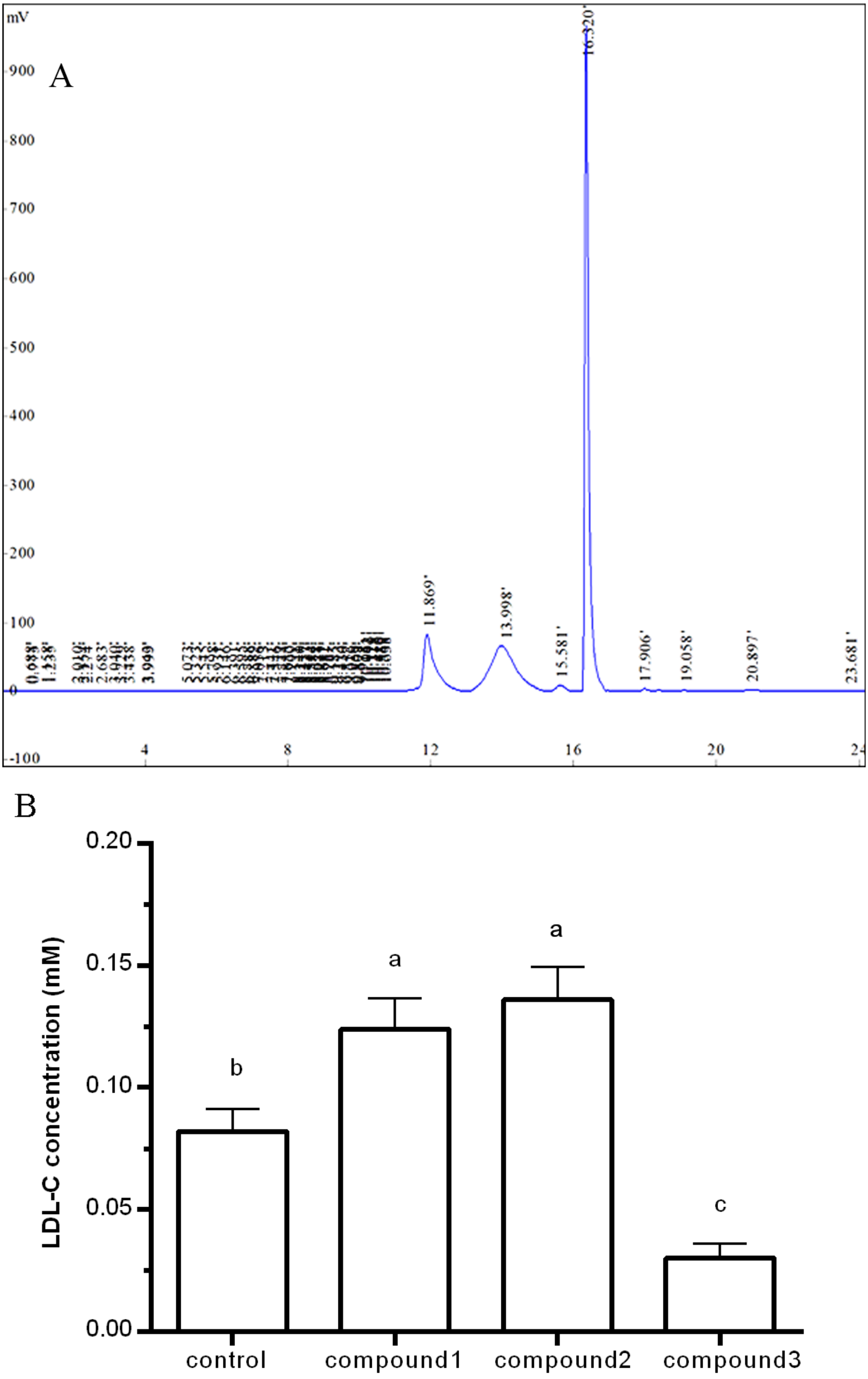
A
mV
900
800
700
600
500
400
300
200
100
0
-100
4
8
12
16
20
11.869'
13.998'
15.581'
16.320'
17.906'
19.058'
20.897'
23.681'
B
LDL-C concentration (mM)
0.20
0.15
0.10
0.05
0.00
b
a
a
c
control
compound1
compound2
compound3

Figure S1. The purification, anti-cholesterol activity of the saponin from *Thelenota ananas*. (A) The semi-preparative HPLC chromatogram of the purified saponins. (B) The anti-cholesterol activity of the compounds separated by semi-preparative HPLC.

Original full-length blots

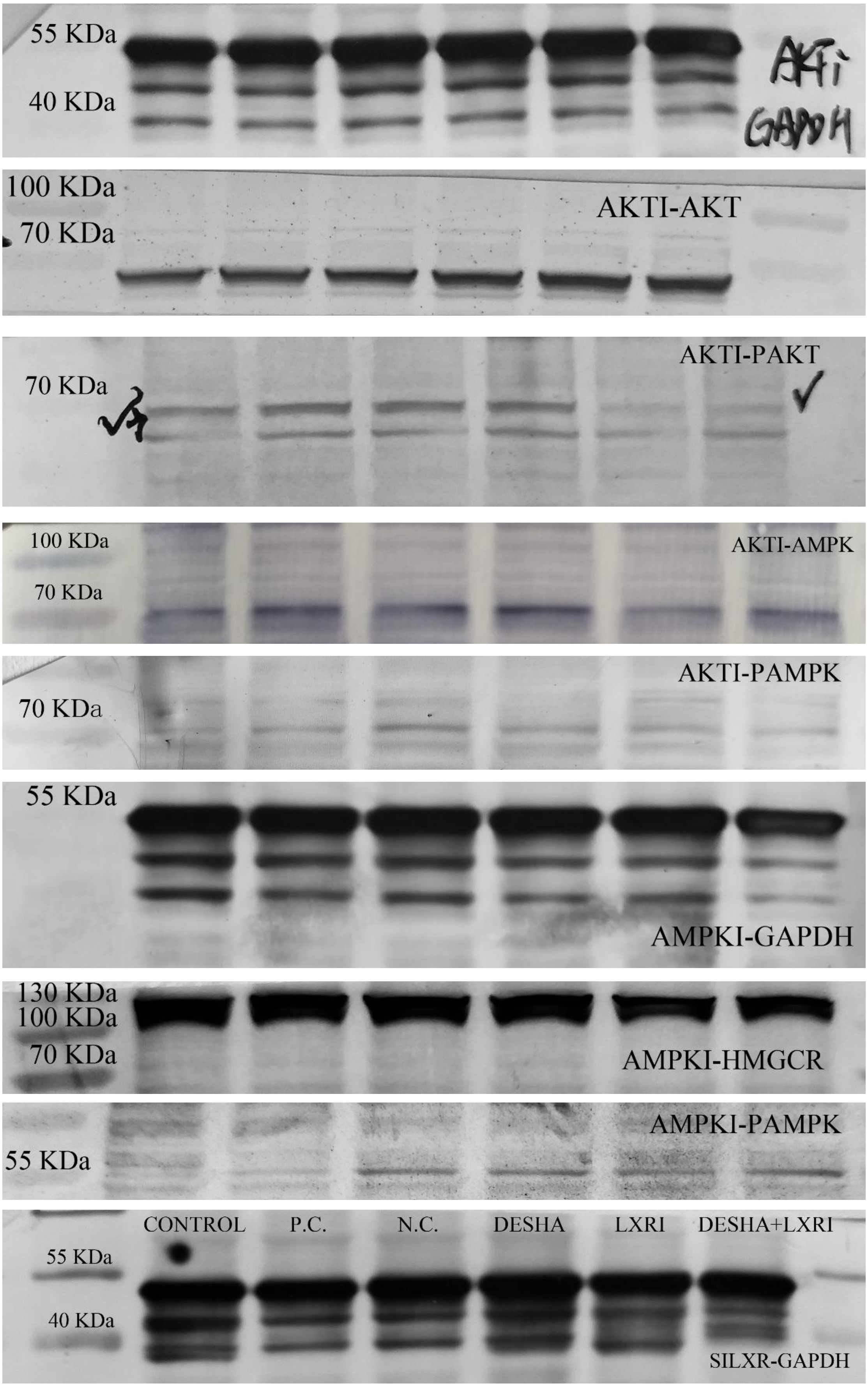

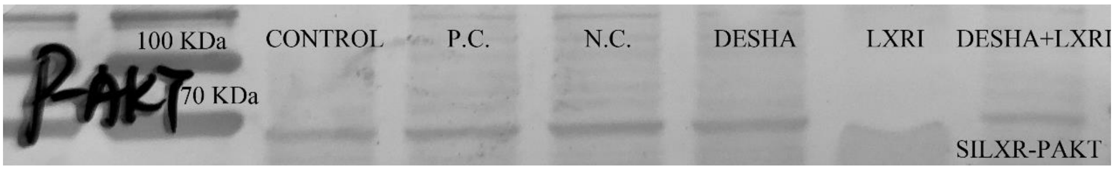
PAKT
100 KDa
70 KDa
CONTROL
P.C.
N.C.
DESHA
LXRI
DESHA+LXRI
SILXR-PAKT
